# Pancreatic cancer challenge in 52 Asian countries: age-centric insights and the role of modifiable risk factors (1990-2019)

**DOI:** 10.3389/fonc.2023.1271370

**Published:** 2023-10-02

**Authors:** Xin Xiang, Xuejie Chen, Yue He, Yiwei Wang, Weitong Xia, Shuyu Ye, Sidan Wang, Yi Xiao, Quansi Li, Xiaoyan Wang, Weiwei Luo, Jingbo Li

**Affiliations:** Department of Gastroenterology Changsha, Central South University Third Xiangya Hospital, Hunan, China

**Keywords:** global burden of disease, pancreatic cancer, Asia, public health, demographic aging

## Abstract

**Background:**

Pancreatic cancer is renowned for its elevated incidence and mortality rates on a global scale. The disease burden of pancreatic cancer is anticipated to increase, particularly in Asia, due to its vast and rapidly aging population.

**Methods:**

Data from the Global Burden of Disease 2019 were analyzed for pancreatic cancer burden across 52 countries in Asia, including the incidence, mortality, and disability-adjusted life years (DALY) for pancreatic cancer, with a focus on risk factors such as high body mass index (BMI), elevated fasting plasma glucose, and smoking. We applied the Estimated Annual Percentage Change, the Age–Period–Cohort model, and decomposition analysis to evaluate incidence trends and effects.

**Results:**

From 1990 to 2019, both incidence and mortality rates of pancreatic cancer in Asia significantly increased, with an average annual standardized incidence rate change of 1.73%. Males consistently exhibited higher rates than females, with smoking as a key risk factor. Central Asia reported the highest rates, and South Asia the lowest. The incidence rose with age, peaking in those aged 70~74. The disease burden increased in all age groups, particularly in populations aged 55 and above, representing 84.41% of total cases in 2019, up from 79.01% in 1990. Pancreatic cancer ranked the fifth in incidence among six major gastrointestinal tumors but presented a significant growth rate of mortality and DALY.

**Conclusion:**

With the growing, aging population in Asia, the pancreatic cancer burden is projected to escalate, bringing a significant public health challenge. Hence, comprehensive public health strategies emphasizing early detection, risk modification, and optimized treatment of pancreatic cancer are imperative.

## 1 Introduction

Pancreatic cancer ranks as the twelfth most prevalent cancer worldwide and stands as the seventh leading cause of death associated with cancer (1). Regarding histological classifications, exocrine tumors account for 95% of pancreatic cancer, predominantly presenting as pancreatic ductal adenocarcinoma. The residual fraction primarily includes endocrine pancreatic cancer, which progress more slowly and usually with favorable prognosis (2). Pancreatic cancer is extremely invasive. Owing to the lack of distinct clinical symptoms and early-stage diagnostic tools, many patients are only diagnosed during the advanced stages of the disease, which leads to a bleak prognosis and increased mortality. The cumulative five-year survival rate is roughly around 10% (3–5). Although multidisciplinary care and adjuvant chemotherapy can improve the prognosis, early surgical resection remains the only chance for cure (6).

Studies have shown that the age-standardized mortality rates are highest in high-income areas, such as North America and Western Europe (7). In Asia, the pancreatic cancer situation has unique aspects, although its burden is generally lower contrasted with affluent Western nations, the growth rate in Asia is accelerating (7). Lifestyle, dietary habits, and genetic factors might be associated with the epidemiological features of pancreatic cancer. Firstly, smoking stands as one of the most consequential risk contributors to pancreatic cancer (8–10). Secondly, a heightened body mass index (BMI) correlates with a greater chance of developing pancreatic cancer (11–13). Thirdly, elevated fasting glucose levels, especially in type 2 diabetes, is a significant risk determinant for pancreatic cancer (14–16). In addition, factors such as alcohol consumption, physical inactivity, hypertension, and hypercholesterolemia contribute to the development of pancreatic cancer, which may exacerbate its burden in Asia (17–19).

Furthermore, the population of Asia accounts for nearly 60% of the global population. With the aging population, the cases of pancreatic cancer is expected to increase (20, 21). Despite this, comprehensive research on the epidemiology of pancreatic cancer in Asia remains scarce. Therefore, an in-depth examination of the epidemiological landscape of pancreatic cancer across Asia, especially focusing on the middle-aged and elderly high-risk populations is necessary. This analysis will contribute to a better understanding of the epidemiological characteristics of pancreatic cancer, thereby providing a scientific basis for its effective prevention and control strategies.

## 2 Method

### 2.1 Data source

In our investigation, we specifically focused on the burden of pancreatic cancer across 52 Asian countries. The data was extracted from the Global Burden of Disease Study 2019 (22). To accurately appraise the burden of pancreatic cancer in Asia, we retrieved data specific to this region from the GBD 2019 study (accessible via https://vizhub.healthdata.org/gbd-results/). The data encompassed the incidence, mortality, and DALY associated with pancreatic cancer, coupled with the death toll and DALY related to diverse risk factors spanning from 1990 to 2019. Data were divided by sex, age, and regional location and then aggregated by age across consecutive five-year intervals.

Furthermore, we garnered age-standardized incidence rates (ASIR), mortality rates (ASMR), DALY rates (ASDR), and annual average rates of change in ASDR for the six principal gastrointestinal cancers from 1990 to 2019. This group of cancers encompasses pancreatic, colorectal, liver, esophageal, gastric, as well as gallbladder and biliary tract cancers.

### 2.2 Risk factors

In our study, we incorporated three recognized risk factors for pancreatic cancer - high body mass index (BMI), high fasting plasma glucose, and smoking. We employed a comparative risk evaluation framework to gauge the proportion of deaths and DALYs attributable to these identified risk factors (18, 23).

### 2.3 Estimated annual percentage change

For analyzing the trend of pancreatic cancer incidence, we applied ASIR, ASMR, AMDR, and estimated annual percentage change (EAPC) (24). EAPC is a widely utilized metric to quantify the variations in ASIR over a specified timeframe (25).

### 2.4 Age-period-cohort analysis

The Age–Period–Cohort (APC) model, frequently utilized in epidemiology, was employed to assess the age, period, and cohort effects on pancreatic cancer incidence trends (26). This analysis facilitates the exploration of underlying physiological, social, historical, and environmental factors. Period effects epitomize the fluctuations in incidence over time, while cohort effects relate to changes within specific groups sharing the same birth year. Two pivotal parameters within APC models are the net and local drifts. The net drift highlights the annual percentage alteration in incidence with the passage of time, while local drifts illustrate the yearly percentage changes in incidence within distinct age groups over the same period (27).

### 2.5 Decomposition

Decomposition analysis was employed in our study to estimate the contribution of each factor to the overall incidence of pancreatic cancer (28). The three factors incorporated in this analysis included age structure, population growth, and epidemiological changes.

## 3 Result

### 3.1 The burden of pancreatic cancer and its temporal trends across Asia

**Figure 1:**
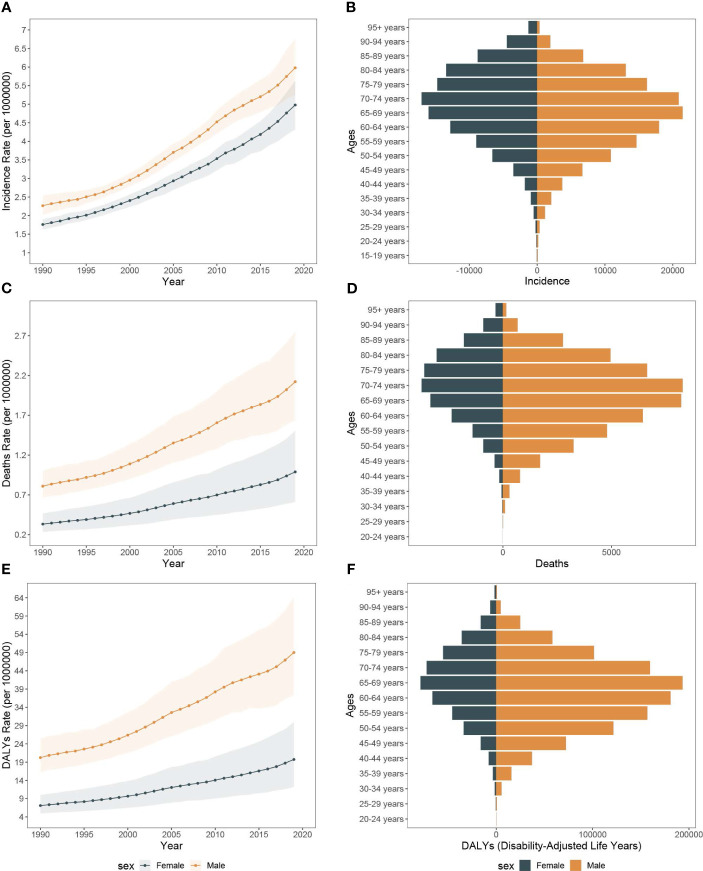
The incidence rate, mortality rate, and DALY rate of pancreatic cancer in Asia from 1990 to 2019 and the counts of incidence, deaths, and DALY of pancreatic cancer across age groups, 2019. **(A)** Trends of pancreatic cancer incidence rate in Asia, 1990-2019. **(B)** The counts of pancreatic cancer new cases across age groups, 2019. **(C)** Trends of pancreatic cancer mortality rate in Asia, 1990-2019. **(D)** The counts of deaths due to pancreatic cancer across age groups, 2019. **(E)** Trends of pancreatic cancer DALY rate in Asia, 1990-2019. **(F)** The counts of DALY due to pancreatic cancer across age groups, 2019.

The years 1990 to 2019witnessed notable rise in the incidence of pancreatic cancer across Asia. Specifically, the newly diagnosed cases grew from 64,444.1 (95% UI, 59,818.7 to 69,270.9) to 249,961.4 (95% UI, 224,066.6 to 274,321.1). Concurrently, the incidence rate of pancreatic cancer rose from 2.0 per 100,000 people (95% CI, 1.9 to 2.2) to 5.5 per 100,000 people (95% CI, 4.9 to 6.0) from 1990 to 2019. Moreover, in this span, the incidence persistently showed a higher trend in males compared to females. In 2019, male cases amounted to 138,552.4 (95% UI, 121,442.8 to 156,604.2), whereas female cases numbered 111,408.96 (95% UI, 96,616.4 to 125,983.5). The incidence rate for 2019 was 6.0 per 100,000 for males (95% CI, 5.2 to 6.8) and 5.0 per 100,000 for females (95% CI, 4.3 to 5.6). Interestingly, peak incidence varied between genders, with the highest incidence observed at 65-69 years in males and 70-74 years in females (**Figures 1A, B**).

**Figure 2:**
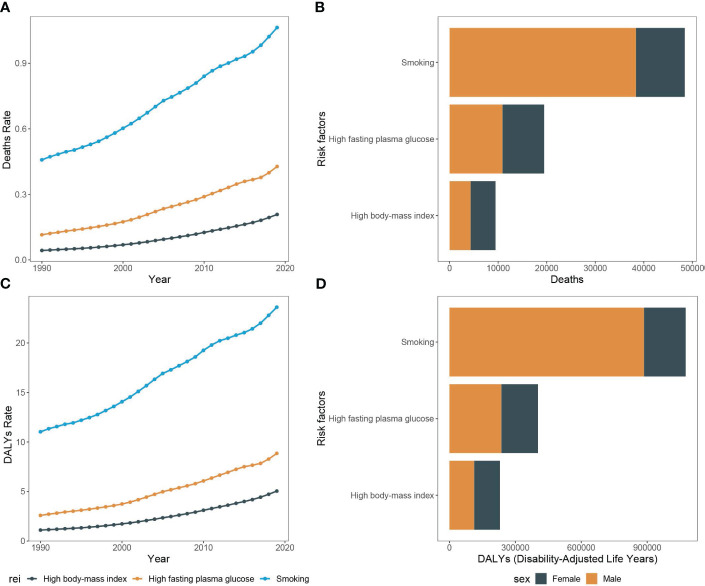
Mortality rate and DALY rate of pancreatic cancer due to smoking, high fasting plasma glucose, and high body-mass index in Asia, 1990-2019 and the counts of deaths and DALY of pancreatic cancer due to smoking, high fasting plasma glucose, and high body-mass index, 2019. **(A)** Trends of pancreatic cancer mortality rate due to smoking, high fasting plasma glucose, and high body-mass index in Asia, 1990-2019. **(B)** The counts of pancreatic cancer deaths due to smoking, high fasting plasma glucose, and high body-mass index, 2019. **(C)** Trends of pancreatic cancer DALY rate due to smoking, high fasting plasma glucose, and high body-mass index in Asia, 1990-2019. **(D)** The counts of pancreatic cancer DALY due to smoking, high fasting plasma glucose, and high body-mass index, 2019.

Between 1990 and 2019, there was a considerable increase in the count of pancreatic cancer-related mortality across Asia. The number rose from 18,421.1 deaths (UI, 15,007.2 to 23,061.4) in 1990 to 71,302.7 deaths (95% UI, 53,637.6 to 94,336.9) in 2019. Concurrently, the mortality rate escalated from 0.6 per 100,000 people (95% CI, 0.5 to 0.7) in 1990 to 1.6 per 100,000 people (95% CI, 1.2 to 2.1) in 2019. Moreover, among the risk factors for pancreatic cancer in Asia, smoking was associated with the highest deaths, accounting for 48,434.5 deaths (95% UI, 40,472.4 to 56,857.7). This was followed by high fasting glucose, which was related to 19,476.7 deaths (95% UI, 4,434.7 to 42,833.9), and high body mass index, which was associated with 9,472.7 deaths (95% UI, 3,059.4 to 19,776.4). Significantly, the number of pancreatic cancer deaths attributable to smoking was greater in males compared to females, with 38,383.6 versus 10,050.9, respectively. In contrast, deaths associated with high body mass index were higher in females than in males, with counts of 5,125.9 and 4,346.8, respectively. The peak age of mortality was consistent in both genders, observed in 70-74 years (**Figures 1C, D**, **2A, B**).

Throughout the study period, the DALY associated with pancreatic cancer demonstrated a significant rise, with 441,609.0 (95% UI, 355,797.4 to 559,679.9) in 1990 to 1,493,534.0 (95% UI, 1,138,083.1 to 1,938,562.2) in 2019. Concurrently, the DALY rate also escalated from 13.8 per 100,000 people (95% CI, 11.2 to 17.5) in 1990 to 34.6 per 100,000 people (95% CI, 26.3 to 45.8) in 2019. In alignment with mortality data, smoking-related DALY for pancreatic cancer was the most significant among the three risk factors evaluated, accounting for 1,075,165.6 DALY (95% UI, 884,311.4 to 1,281,377.2) in the study period. This was followed by high fasting blood glucose at 403,149.9 DALY (95% UI, 90,054.3 to 893,922.9), and finally, high body mass index at 229,781.3 DALY (95% UI, 73,342.4 to 485,585.1). DALY tied to smoking-induced pancreatic cancer were found to be more prevalent in males as compared to females, with counts of 189,368.3 versus 887,797.2, respectively. The age group with highest prevalence was 65-69 years old (**Figures 1E, F**, **2C, D**).

In 2019, Central Asia exhibited the highest ASIR of pancreatic cancer (5.8 per 100,000), ASMR (1.8 per 100,000), and ASDR (41.2 per 100,000). In contrast, South Asia reported the lowest ASIR (2.9 per 100,000), ASMR (0.7 per 100,000), and ASDR (15.0 per 100,000). At the country level, Palau recorded the highest ASIR of pancreatic cancer in Asia (11.4 per 100,000) in 2019, followed by Japan (10.2 per 100,000). Papua New Guinea reported the lowest ASIR (1.7 per 100,000). With regard to ASMR and ASDR, the Palau Islands ranked the highest (3.9 per 100,000 and 84.6 per 100,000, respectively), with Armenia in the second position (3.6 per 100,000 and 81.0 per 100,000, respectively). The lowest ASMR was found in Papua New Guinea (0.5 per 100,000), whereas Bangladesh reported the lowest ASDR (11.2 per 100,000) (**Appendix Figures 1A, C, E**, **Appendix Tables 1**–**3**).

### 3.2 Unveiling the link between tobacco consumption and pancreatic cancer burden in Asia

**Figure 3:**
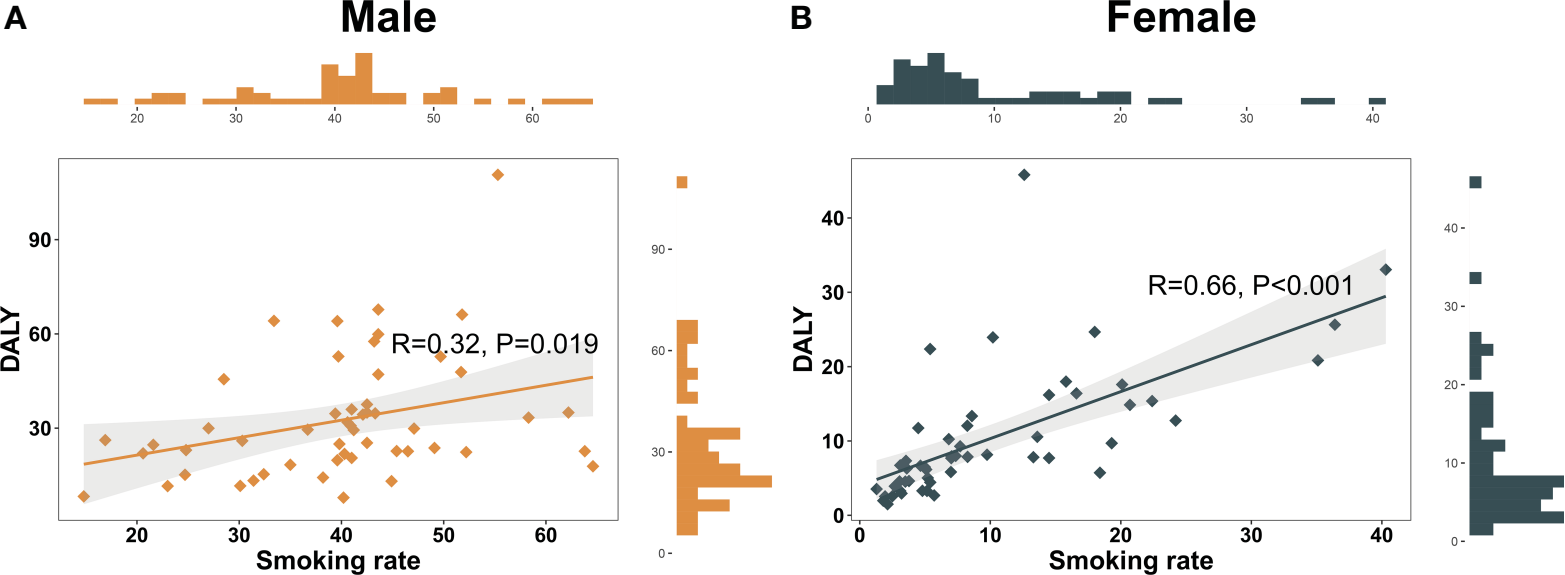
Nomogram of association between smoking prevalence and pancreatic cancer burden in Asian in 2019. **(A)** Nomogram of association between smoking prevalence and pancreatic cancer burden in Asian males in 2019. **(B)** Nomogram of association between smoking prevalence and pancreatic cancer burden in Asian females in 2019.

In our investigation, tobacco consumption was a crucial contributor to the substantial burden of pancreatic cancer in Asia. By employing Pearson’s correlation analysis, a significant association between smoking prevalence and smoking-attributable DALY rate was uncovered in both genders (Male: R = 0.32, P = 0.019; Female: R = 0.66, P < 0.001) (**Figures 3A, B**). In certain nations, such as Armenia and Georgia, male smoking prevalence surpasses 50 per 100,000 individuals. These nations also report higher smoking-attributable DALY rates. Specifically, smoking-attributable DALY rates in Armenia and Georgia were recorded as 110.6 per 100,000 and 66.1 per 100,000, respectively. Contrarily, in regions with modest smoking prevalence, such as Bangladesh, the smoking-attributable DALY rate was relatively lower. Despite a smoking prevalence of 44.9 per 100,000 among Bangladeshi males, the smoking-attributable DALY rate was only 13.1 per 100,000 (**Appendix Figures 2A–D**).

From gender perspective, female smoking prevalence remains low in most Asian countries. However, a substantial association between smoking prevalence and smoking-attributable DALY rates exists among females in certain regions. For instance, in the Federated States of Micronesia, female smoking prevalence was 36.4 per 100,000, with a corresponding smoking-attributable DALY rate of 34.9 per 100,000 (**Appendix Figure 2D**).

### 3.3 EAPC analysis of pancreatic cancer from 1990 to 2019

Between 1990 and 2019, the ASIR and ASMR and ASDR of pancreatic cancer demonstrated varied trends across Asian nations. The overall estimated AAPC for the ASIR stood at 1.73%. Remarkably, the ASIR witnessed the most significant increase in Kazakhstan, with an EAPC of 7.66 (95% CI: 6.53 to 8.81), followed by Uzbekistan with an EAPC of 4.41 (95% CI: 4.19 to 4.63), and Vietnam with an EAPC of 3.82 (95% CI: 3.59 to 4.04). On the contrary, Thailand and South Korea presented relatively stable incidence rates, with EAPCs of 0.04 (95% CI: -0.26 to 0.33) and 0.03 (95% CI: -0.21 to 0.26), respectively. Interestingly, Samoa demonstrated a significant decrease in incidence rates with an EAPC of -0.35 (95% CI: -0.59 to -0.11) (**Appendix Figure 1B**).

Regarding ASMR and ASMR rates, the most considerable increases were reported in Kazakhstan, Uzbekistan, and Vietnam. Kazakhstan registered the highest EAPC in both mortality (8.32, 95% CI: 7.21 to 9.45) and DALY rates (8.56, 95% CI: 7.34 to 9.79). Uzbekistan reported an EAPC of 5.81 (95% CI: 5.60 to 6.02) for mortality and 5.19 (95% CI: 5.02 to 5.37) for DALY rates. Concurrently, Vietnam recorded an EAPC of 4.32 (95% CI: 4.05 to 4.59) for mortality and 4.12 (95% CI: 3.86 to 4.38) for DALY rates. In contrast, countries including Singapore, Thailand, Japan, and South Korea showed decrease in both mortality and DALY rates, with Japan registering the most significant decline in mortality (EAPC = -0.50, 95% CI: -0.61 to -0.40) and DALY rates (EAPC = -0.55, 95% CI: -0.66 to -0.45) (**Appendix Figures 1D, F**).

### 3.4 Divergent trends in pancreatic cancer incidence by APC analysis

**Figure 4:**
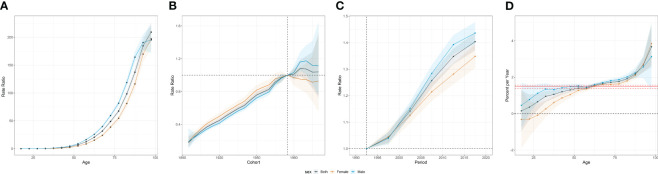
Age-period-cohort effects of incidence from 1990 to 2019 in Asia. **(A)** Age effects are represented by the fitted longitudinal age curves of incidence (per 100,000 person-years) adjusted for period deviations. **(B)** Cohort effects are represented by the relative risk of prevalence (prevalence rate ratio) and computed as the ratio of age-specific rates in each cohort compared to the referent 1975 cohort. **(C)** Period effects are represented by the relative risk of prevalence (prevalence rate ratio) and computed as the ratio of age-specific rates in each period compared to the referent 1990-1995 period. **(D)** Local drifts indicate the annual percentage change of prevalence (% per year) across five-year age groups (from 0 to 4 to 95 plus years). The shaded areas indicate the corresponding 95% CIs of each point estimate.

In the APC analysis, we noted divergent trends in pancreatic cancer incidence between genders. The incidence of pancreatic cancer escalates with age, peaking among individuals aged 95 years or above. Prior to age 55, the incidence remains relatively low, but experiences an exponential rise thereafter. This increase is particularly pronounced among the elderly population (**Figure 4A**).

On a global scale, younger birth cohorts displayed an overall incline in the risk of pancreatic cancer. Interestingly, the effect of the birth cohort varied by gender. Prior to 1975, females encountered a more significant increase in risk compared to males. However, post-1975, the birth cohort effect was more conspicuous among males (**Figure 4B**).

Upon investigating the period effect, we identified a steady upsurge in the risk of pancreatic cancer throughout the study duration in Asia. Notably, males demonstrated a higher risk relative to their female counterparts (**Figure 4C**).

Within Asia, the average annual incidence rate of pancreatic cancer escalated across all age brackets. Yet, a decline in incidence was observed in females under the age of 35, representing a notable exception. Among individuals aged 95 years or more, a considerable surge in incidence was witnessed, with an AAPC of 3.85% (95% CI: 2.69 to 5.02). This translates to a striking 115.5% augmentation over the last three decades (**Figure 4D**).

### 3.5 Deciphering the impact of population growth, aging, and epidemiological changes on pancreatic cancer in Asia: a decomposition analysis

**Figure 5:**
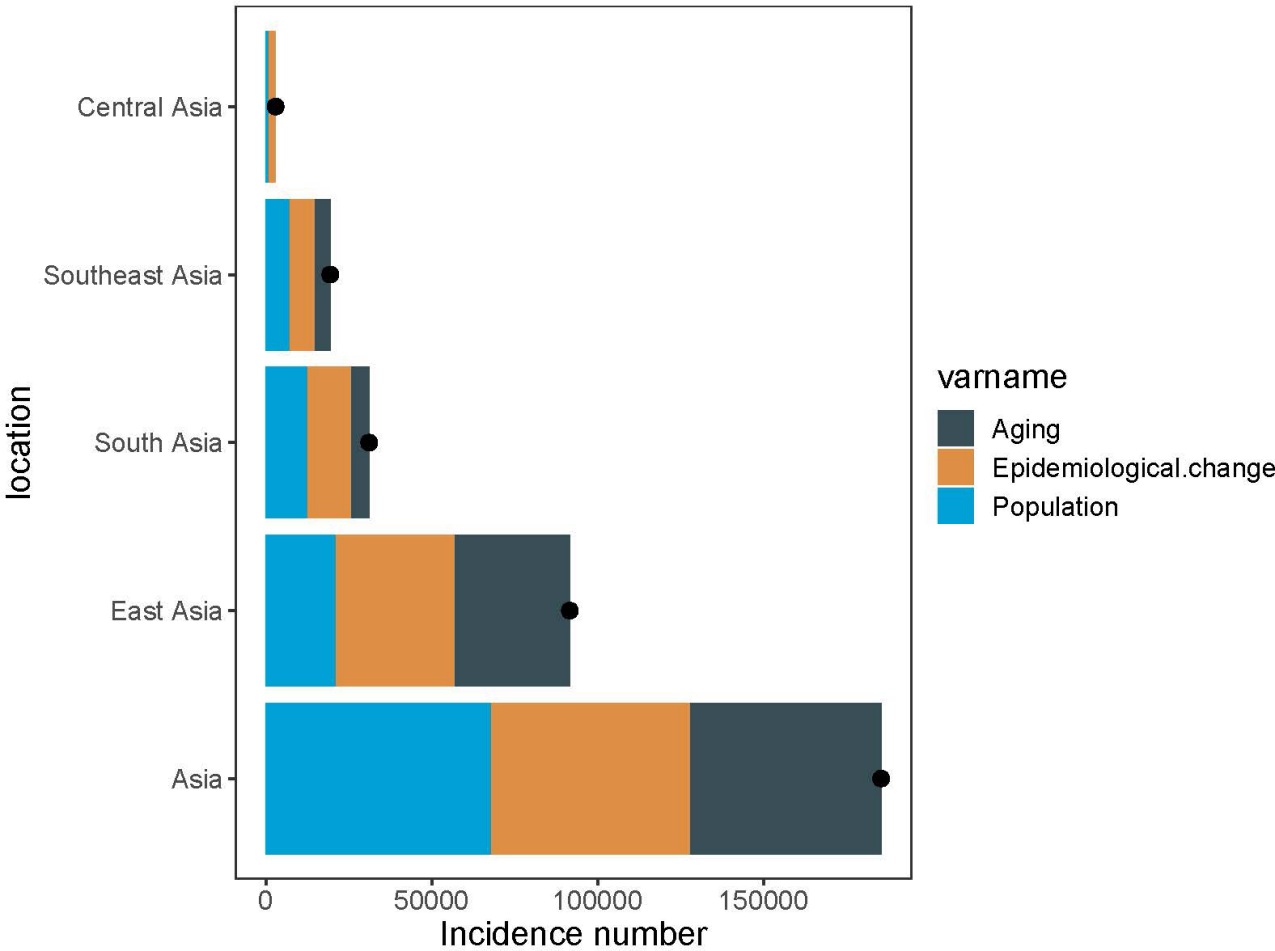
Changes in pancreatic cancer incidence according to population-level determinants of population growth, aging, and epidemiological change from 1990 to 2019. The black dot represents the overall value of change contributed by all three components. For each component, the magnitude of a positive value indicates a corresponding increase attributed to the component; the magnitude of a negative value indicates a corresponding decrease attributed to the related component.

The findings revealed a significant increase in pancreatic cancer incidence throughout Asia, with the most marked increase in South and East Asia. In Asia as a whole, population growth accounted for 36.6% of the overall increase in pancreatic cancer cases from 1990 to 2019, highlighting the population dynamics on disease burden. Additionally, population aging contributed significantly, with 31.1% to the overall increase in cases. This effect was most evident in East Asia (38.0%), followed by South Asia (17.9%) and Southeast Asia (24.2%). Contrarily, the influence of aging on the rise of pancreatic cancer incidence was subdued in Central Asia, only contributing to 3.5% of the total upsurge. The increase in pancreatic cancer cases in Central Asia was primarily driven by the rising incidence, constituting 63.5% of the overall change over the past 30 years (**Figure 5**).

### 3.6 Pancreatic cancer trends in individuals aged 55 and above

**Figure 6:**
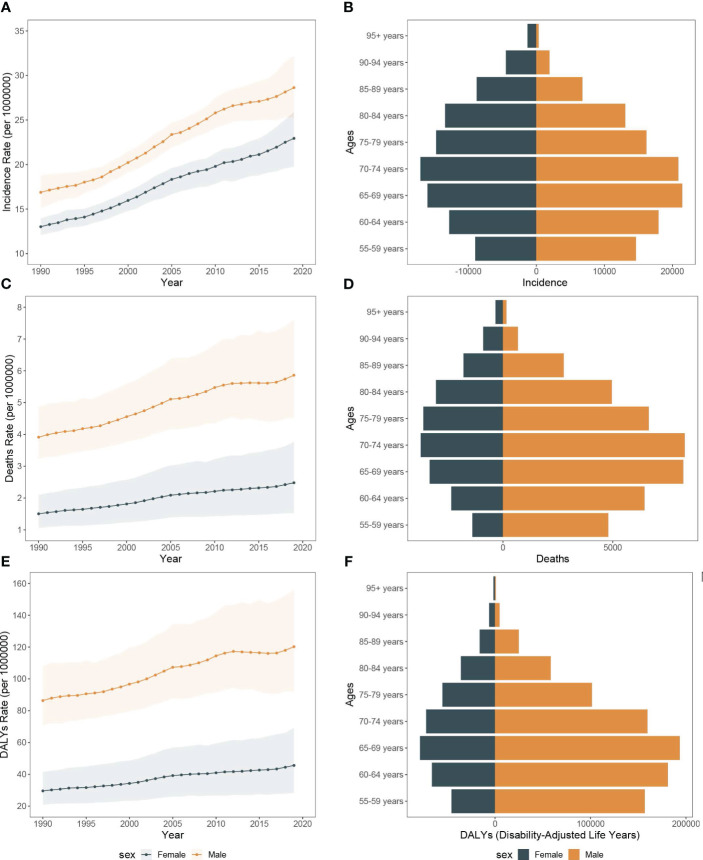
The incidence rate, mortality rate, and DALY rate of pancreatic cancer among people over 55 years in Asia from 1990 to 2019 and the counts of incidence, deaths, and DALY of pancreatic cancer across age groups, 2019. **(A)** Trends of pancreatic cancer among people over 55 years incidence rate in Asia, 1990-2019. **(B)** The counts of pancreatic cancer new cases across age groups among people over 55 years, 2019. **(C)** Trends of pancreatic cancer among people over 55 years mortality rate in Asia, 1990-2019. **(D)** The counts of deaths due to pancreatic cancer across age groups among people over 55 years, 2019. **(E)** Trends of pancreatic cancer among people over 55 years DALY rate in Asia, 1990-2019. **(F)** The counts of DALY due to pancreatic cancer across age groups among people over 55 years, 2019.

Based on our analysis, the burden of pancreatic cancer in Asia has increased significantly, particularly among middle-aged and elderly individuals aged 55 and older. Between 1990 and 2019, the number of pancreatic cancer patients aged 55 and older in Asia increased from 50,975.0 (95%UI, 47,496.9 to 54,333.9) to 210,992.2 (95%UI, 188,959.3 to 230,749.0). The proportion of this age group within the total population rose from 79.01% in 1990 to 84.41% in 2019. Moreover, the incidence of pancreatic cancer in the elderly aged 55 years and older increased from 14.9 (95%CI, 13.9 to 15.9) per 100,000 population in 1990 to 25.7 (95%CI, 23.0 to 28.1) in 2019, which was nearly five times higher than that of the overall age group. Mirroring the trend observed in all age groups, the incidence of pancreatic cancer in individuals over 55 years old was higher in males than females in Asia (**Figures 6A, B**).

**Figure 7:**
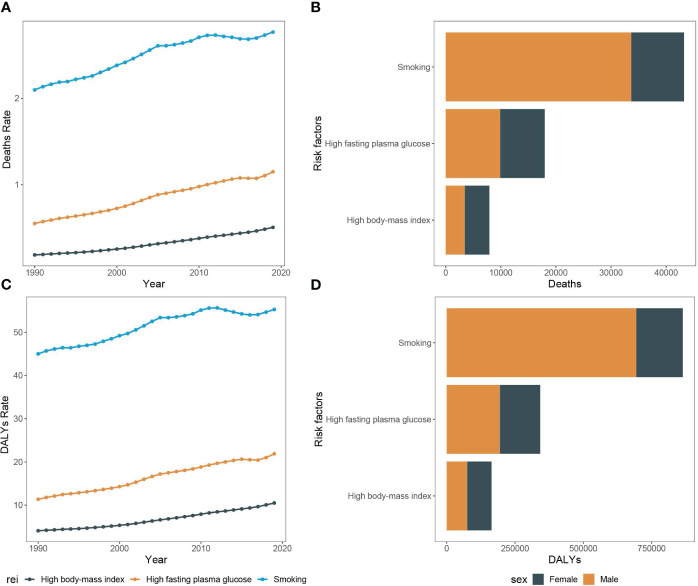
Mortality rate and DALY rate of pancreatic cancer among people over 55 years due to smoking, high fasting plasma glucose, and high body-mass index in Asia, 1990-2019 and the counts of deaths and DALY of pancreatic cancer among people over 55 years due to smoking, high fasting plasma glucose, and high body-mass index, 2019. **(A)** Trends of pancreatic cancer among people over 55 years mortality rate due to smoking, high fasting plasma glucose, and high body-mass index in Asia, 1990-2019. **(B)** The counts of pancreatic cancer deaths due to smoking, high fasting plasma glucose, and high body-mass index among people over 55 years, 2019. **(C)** Trends of pancreatic cancer among people over 55 years DALY rate due to smoking, high fasting plasma glucose, and high body-mass index in Asia, 1990-2019. **(D)** The counts of pancreatic cancer DALY due to smoking, high fasting plasma glucose, and high body-mass index among people over 55 years, 2019.

From 1990 to 2019, the number of all-risk factors deaths due to pancreatic cancer in Asia increased from 15,994.7 (95%UI, 13,036.4 to 19,977.5) to 63,557.0 (95%UI, 47,851.5 to 84,228.1), and the mortality rates increased from 2.7 (95%CI, 2.2 to 3.3) to 4.1 (95%CI, 3.1 to 5.4). Additionally, among the middle-aged and elderly individuals over 55 years old, smoking-related pancreatic cancer deaths accounted for the largest proportion (68.0%), followed by high fasting blood glucose (28.3%), with high body mass index representing the lowest (12.5%). Over the study period, the all-cause disability-adjusted years from pancreatic cancer increased from 341,425.4 (95%UI, 277,439.9 to 427,972.0) in 1990 to 1,259,809.6 (95%UI, 953,019.8 to 1,664,981.1) in 2019. The disability-adjusted age rates also increased from 56.5 (95%CI, 45.9 to 70.8) in 1990 to 80.6 (95%CI, 61.0 to 106.5) in 2019 (**Figures 6C–F**, **7A–D**).

### 3.7 Comparative analysis of pancreatic cancer and other major gastrointestinal cancers among individuals aged 55 and above in Asia

**Figure 8:**
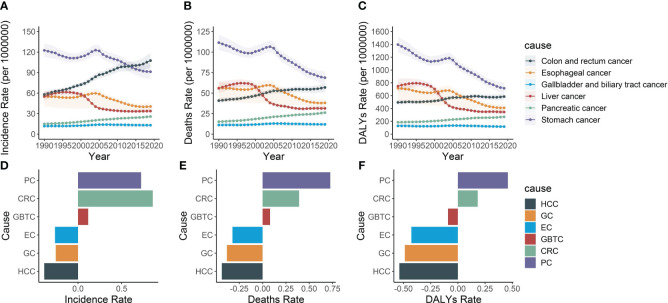
The incidence rate, mortality rate, and DALY rate of six gastrointestinal cancers and their average annual percentage change from 1990 to 2019. **(A)** Trends in the incidence rate of pancreatic, colon and rectum, liver, stomach, gallbladder and biliary tract and esophageal cancers in Asia, 1990-2019. **(B)** Trends in the mortality rate of pancreatic, colon and rectum, liver, stomach, gallbladder and biliary tract and esophageal cancers in Asia, 1990-2019. **(C)** Trends in the DALY rate of pancreatic, colon and rectum, liver, stomach, gallbladder and biliary tract and esophageal cancers in Asia, 1990-2019. **(D)** Average annual incidence rates of pancreatic, colon and rectum, liver, stomach, gallbladder and biliary tract and esophageal cancers in Asia, 1990-2019. **(E)** Average annual mortality rates of pancreatic, colon and rectum, liver, stomach, gallbladder and biliary tract and esophageal cancers in Asia, 1990-2019. **(F)** Average annual DALY rates of pancreatic, colon and rectum, liver, stomach, gallbladder and biliary tract and esophageal cancers in Asia, 1990-2019. PC, Pancreatic cancer; CRC, Colon and rectum cancer; GBTC, Gallbladder and biliary tract cancer; EC, Esophageal cancer; GC, Stomach cancer; HCC, Liver cancer.

Consistently over the span of the past three decades, pancreatic cancer has maintained its rank as the fifth most common in incidence. In 1990, the cases of pancreatic cancer were 210,992.2, accounting for 8.2% of the six major gastrointestinal tumors. Among these cancers, gastric cancer remained the most prevalent digestive cancer in 2019, comprising 29.3% of all tumors, despite its incidence declining since 1990. Colorectal cancer exhibited a continuous rise in incidence throughout the study period and surpassed esophageal and liver cancers, becoming the second most common cancer in 2019. Conversely, gallbladder and biliary tract cancer exhibited the lowest incidence, without significant changes observed during the period (**Figures 8A, D**).

Regarding mortality and DALY, pancreatic cancer ranked the fifth among the six digestive tract diseases. Throughout the study period, the burden of gastric, esophageal, and liver cancers declined, while the burden of colorectal, pancreatic, gallbladder, and biliary tract cancers increased among individuals aged 55 and over. However, pancreatic cancer exhibited the most rapidly increasing mortality rates (0.7, 95% CI, 0.5 to 0.9) and DALY rates (0.5, 95% CI, 0.3 to 0.7). Conversely, esophageal cancer, gastric cancer, and liver cancer demonstrated a downward trend in both mortality and DALY rates, with liver cancer exhibiting the steepest decline at -0.4 (95% CI, -0.5 to -0.3) and -0.5 (95% CI, -0.6 to -0.5), respectively (**Figures 8B–F**).

## 4 Discussion

In the past few decades, the global burden of pancreatic cancer continuously increased, with a particularly significant surge in Asia. Notwithstanding, research concerning pancreatic cancer remains concentrated in a few developed Asian countries such as China, Japan, and South Korea. This research combines the data from the Global Burden of Disease Study 2019 and provide a comprehensive analysis of the impact of pancreatic cancer in 52 countries in Asia.

Between 1990 and 2019, the incidence, mortality, and DALY rates for pancreatic cancer increased significantly in Asia, irrespective of age and sex adjustments. These results signify a rising burden of pancreatic cancer in the past thirty years. Notably, the disease burden was consistently higher in males compared to females during the study period, with an earlier age of onset observed in male. On a global scale, the incidence of pancreatic cancer typically presents a slight male predominance, particularly in individuals under the age of 75 (7, 29, 30). This gender disparity may be attributed to factors such as smoking and physiological differences. Assessing various risk factors, we discovered that the burden of pancreatic cancer attributable to smoking was considerably higher in male than in female. Conversely, the burden ascribed to high BMI and high fasting blood glucose was comparable between male and female. This suggests that while smoking plays a more significant role in male, the impact of high BMI and high fasting blood glucose is consistent in both genders. Existing studies have confirmed that the risk of pancreatic cancer in smokers is twice that of non-smokers (31, 32). Individuals in the BMI range of 25 to 30 and 30 to 35 kg/m2 showed a heightened risk of pancreatic cancer by 13% and 19% respectively, when contrasted with those with normal BMIs (33).

Upon examination of the three risk factors, smoking emerged as the predominant factor contributing to the increased burden of pancreatic cancer in Asia. We discerned a linear relationship between smoking prevalence and pancreatic cancer burden, with regions of higher smoking prevalence typically exhibiting a greater burden. In Asian, the rate of smoking in male markedly surpasses the female. Research has verified that smoking contribute to the higher risk of diabetes by 44%, as it triggers insulin resistance and inadequate insulin secretion, and amplifies the incidence of abdominal obesity (34). Nonetheless, with the passage of time, female smoking rates are gradually rising, which potentially precipitate a significant escalation in the female-specific burden of pancreatic cancer in the future. Implementing strategies to curtail smoking rates in countries with high smoking prevalence, such as Armenia and Georgia, will reduce the burden of pancreatic cancer. Due to a greater percentage of body fat in Asian population compared to Western ones, the World Health Organization advocates for a lower threshold for obesity in these populations (35). A Korean study, which defined obesity as BMI ≥ 25 kg/m2, showed that obese individuals had a slightly increased risk of pancreatic cancer, but this risk was significantly lower than those reported in the West (36, 37). Research indicates a strong correlation between obesity and diabetes and economic advancement, with higher prevalence of both conditions in more developed regions. With the poverty alleviation in China and other Asian countries, the rates of obesity and diabetes also continue to rise, which may lead to higher prevalence of pancreatic cancer (38, 39).

The incidence and mortality rates of pancreatic cancer have surged notably in a majority of Asian countries. Nevertheless, stark disparities in incidence rates exist in different countries and regions. The ASIR, for instance, exhibited a 6.65-fold difference at its extremes (comparing Palau at 11.43 and Papua New Guinea at 1.72). EAPC analysis revealed that the average annual growth rates of the ASIR in Asia was 1.73%. Countries such as Kazakhstan, Uzbekistan, and Vietnam witnessed the most rapid rise of these rates. In contrast, Thailand and South Korea demonstrated relative stability in their ASIR throughout the study period, with Samoa being the only country exhibiting a declining trend. Moreover, most Asian countries continue to see an upward trajectory in ASMR and ASDR. However, countries including Singapore, Japan, and South Korea show a declining trend. An analysis of pancreatic cancer mortality in East Asia also confirmed that while Japan still has the highest ASMR for pancreatic cancer, it has witnessed a downturn in both males and females after the year 2012, and the average annual percentage change in total population from 2012 to 2019 was -0.63%. South Korea is the second-highest ASMR country for pancreatic cancer, with the mortality continue to decline since 2011. A comparable declining trend was observed exclusively in East Asia, a region marked by a high socio-demographic index (40). This could potentially be ascribed to their advanced economies and superior healthcare systems.

Our findings revealed a distinct age-related increase in pancreatic cancer risk, with greater risk in middle-aged and elderly individuals compared to younger populations. Over the study period, a steady increase in incidence was observed across all age groups, but most prominently among middle-aged and older adults. An APC study in East Asia also confirmed that the risk of death from pancreatic cancer increases with age (40). This increase may be attributed to the rising prevalence of obesity and diabetes, which have been proliferating due to the economic advancement of Asian countries. The annual increase in smoking rates also stands as a crucial factor contributing to this trend.

At continental level, population growth served as the most substantial driver of the increase in pancreatic cancer cases. Currently, more than 4 billion people live in Asia, accounting for 60% of the world’s population. It is predicted that by 2040, the global population will increase by 2.1 billion, of which 1 billion in Asia (41). Closely followed by cases attributable to population aging. South and East Asia experienced the most pronounced rise in incidence, primarily attributable to population growth. In contrast, Central Asia’s increase in cases was largely due to epidemiological transitions, potentially linked to the region’s escalating smoking prevalence. Furthermore, factors such as deficient of prevention measures, limited access to quality healthcare, lack of available treatment options, scarcity of radiotherapy, absence of national cancer strategies, lack of multidisciplinary teams, intricate therapeutic geographies, and the uneven scale of social development might contribute to the increased incidence in Central Asia (42).These findings necessitate further investigation to uncover the factors of these trends and to devise targeted interventions aimed at alleviating the disease’s impact.

In Asia, individuals aged 55 and above bear the brunt of pancreatic cancer’s disease burden. The proportion of cases within this demographic expanded from 79% in 1990 to 84%, signifying an increasingly grave and rising burden of pancreatic cancer in older individuals. This disparity may be attributed to the cumulative exposure to various risk factors for cancer, including long-term smoking, high-fat diets, chronic pancreatitis, and obesity (23, 43, 44). The prolonged accumulation of these risk factors may lead to aberrant growth and carcinogenesis of pancreatic cells (45). Furthermore, age-related physiological changes, such as reduced cellular repair and replacement rates, along with declining immune system capabilities, contribute to the heightened susceptibility of elderly individuals to pancreatic cancer (46). The mortality and DALY rates associated with pancreatic cancer have both exhibited a discernible upward trajectory. With the ongoing demographic aging in Asian countries, particularly in highly populous nations like China and India, the burden of pancreatic cancer is likely to become more severe, potentially leading Asia to have the world’s highest disease burden.

We further compared the pancreatic cancer burden among those aged 55 and above to that of the other five most prevalent gastrointestinal (GI) cancers, namely, gastric, esophageal, liver, colorectal, and gallbladder and biliary tract cancers. Although pancreatic cancer mortality and DALY did not reach the highest levels compared to other GI cancers, but the mortality and DALY rate of pancreatic cancer showed a most rapidly increase among the six cancers. This surge could be attributed to population aging and changes in lifestyle (47).

While Global Burden of Disease (GBD) studies yield valuable health insights worldwide, they still face inherent limitations. Data quality varies due to inconsistent research design and implementation in different regions, impacting accuracy. One notable limitation is the potential for ecological fallacy, where associations observed at the population level might not reflect those at individual level. Hence, caution should be exercised when making inferences about individual risks based on population-level findings. Moreover, certain regions contribute unreliable or incomplete health data, thereby limiting the comprehensiveness of the GBD. Importantly, our study relied on the GBD’s available data, which meant that we could not incorporate all known risk factors for pancreatic cancer, such as alcohol consumption, due to their absence or lack of robust data in the database. Nonetheless, the 2019 GBD data appears more reliable than of the 2017 edition, possibly due to improved data collection, analytical methods, and overall data quality. Although these advancements are promising, we must cautiously interpret GBD data, considering its inherent limitations, and strive to improve its robustness through ongoing research and refinement (48).

## 5 Conclusion

In conclusion, the burden of pancreatic cancer in Asia, particularly among the elderly population, has been rising over the past three decades. This increase can be attributed to a combination of factors, including population aging, changes in lifestyle behaviors, and epidemiological shifts. The findings highlight the need for tailored interventions and policies targeting these high-risk populations and addressing modifiable risk factors such as smoking and obesity. It also underscores the importance of early detection and management strategies for pancreatic cancer to mitigate its public health impact, especially.

## Data availability statement

The datasets presented in this study can be found in online repositories. The names of the repository/repositories and accession number(s) can be found below: https://ghdx.healthdata.org/gbd-results-tool.

## Author contributions

XX: Conceptualization, Formal Analysis, Methodology, Writing – original draft, Writing – review & editing. XC: Conceptualization, Data curation, Formal Analysis, Methodology, Visualization, Writing – original draft. YH: Formal Analysis, Validation, Writing – review & editing. YW: Writing – original draft, Writing – review & editing. WX: Resources, Writing – review & editing. SY: Writing – review & editing, Resources, Supervision. SW: Writing – review & editing, Visualization. QL: Investigation, Supervision, Writing – review & editing. YX: Data curation, Supervision, Writing – review & editing. XW: Data curation, Resources, Writing – review & editing. WL: Conceptualization, Project administration, Supervision, Writing – original draft, Writing – review & editing. JL: Conceptualization, Funding acquisition, Project administration, Supervision, Writing – review & editing.
