# Supplementary material for: Pancreatic cancer challenge in 52 Asian countries: age-centric insights and the role of modifiable risk factors (1990-2019)
Source: Front Oncol. 2023 Oct 2;13:1271370. doi: 10.3389/fonc.2023.1271370 (PMC10577443; doi:10.3389/fonc.2023.1271370)
Supplement: Supplementary file 1 [file DataSheet_1.docx]

**Supplementary appendix**

**Contents:**

**Appendix Tables**

Appendix Table 1: Pancreatic cancer incidence in 1990 and 2019 for both sexes in age-standardised rates by location

Appendix table 2: Deaths due to pancreatic cancer in 1990 and 2019 for both sexes in age-standardised rates by location

Appendix Table 3: DALYs due to pancreatic cancer in 1990 and 2019 for both sexes in age-standardised rates by location

**Appendix Figures**

Appendix Figure 1: **Map of age-standardized incidence rate, mortality rate, DALY rate of pancreatic cancer in Asia, 2019, and age-standardized Estimated Annual Percentage Change from 1990 to 2019.** (A) Map of age-standardized incidence rate in Asia, 2019. (B) EAPC of age-standardized incidence rate from 1990 to 2019. (C) Map of age-standardized mortality rate in Asia, 2019. (D) EAPC of age-standardized mortality rate from 1990 to 2019. (E) Map of age-standardized DALY rate in Asia, 2019. (F) EAPC of age-standardized DALY rate from 1990 to 2019.

Appendix Figure 2: **Map of the smoking prevalence of pancreatic cancer across sex in Asia, 2019 and age-standardized DALY rate of pancreatic cancer due to smoking, 2019.** (A) Map of smoking prevalence among males in Asia, 2019. (B) Map of smoking prevalence among females in Asia, 2019 (C) Age-standardized DALY rate of pancreatic cancer among males in Asia, 2019. (D) Age-standardized DALY rate of pancreatic cancer among females in Asia, 2019.

**Appendix table 1: Pancreatic cancer incidence in 1990 and 2019 for both sexes in age-standardised rates by location.**

|  | 1990 | | 2019 | |
| --- | --- | --- | --- | --- |
|  | Counts (95% UI) | Rate (95% UI) | Counts (95% UI) | Rate (95% UI) |
| Asia | 64444  (59819, 69271) | 3.42  (3.18, 3.65) | 249961  (224067, 274321) | 5.41  (4.83, 5.94) |
| East Asia | 27929  (24282, 31467) | 3.19  (2.80, 3.57) | 119571  (102244, 138482) | 5.80  (4.97, 6.69) |
| Taiwan (Province of China) | 677  (650, 705) | 4.23  (4.05, 4.41) | 3377  (2618, 4397) | 8.54  (6.64, 11.11) |
| China | 26773  (23130, 30262) | 3.17  (2.77, 3.57) | 114964  (98048, 133708) | 5.78  (4.94, 6.69) |
| Republic of Korea | 2234  (2132, 2357) | 7.65  (7.26, 8.09) | 7982  (6671, 9525) | 8.98  (7.51, 10.71) |
| Japan | 16443  (15600, 16888) | 9.77  (9.23, 10.04) | 40981  (33125, 47875) | 10.69  (8.82, 12.47) |
| Democratic People's Republic of Korea | 478  (362, 629) | 2.89  (2.23, 3.69) | 1229  (917, 1575) | 3.80  (2.88, 4.81) |
| Central Asia | 1157  (1027, 1295) | 2.50  (2.21, 2.83) | 4116  (3761, 4529) | 5.84  (5.33, 6.39) |
| Uzbekistan | 166  (129, 251) | 1.48  (1.14, 2.31) | 849  (709, 1012) | 4.83  (4.12, 5.65) |
| Armenia | 176  (150, 206) | 6.64  (5.62, 7.74) | 422  (351, 503) | 10.16  (8.45, 12.07) |
| Mongolia | 31  (25, 38) | 3.07  (2.50, 3.67) | 138  (106, 179) | 5.97  (4.70, 7.51) |
| Turkmenistan | 25  (23, 28) | 1.37  (1.25, 1.51) | 141  (113, 177) | 3.72  (2.99, 4.64) |
| Kazakhstan | 147  (132, 168) | 1.21  (1.08, 1.38) | 1131  (963, 1313) | 6.53  (5.58, 7.57) |
| Azerbaijan | 170  (149, 200) | 3.42  (2.96, 4.03) | 628  (530, 762) | 7.07  (6.05, 8.58) |
| Tajikistan | 91  (64, 134) | 3.37  (2.32, 4.99) | 218  (178, 269) | 5.18  (4.31, 6.35) |
| Kyrgyzstan | 113  (99, 128) | 3.70  (3.24, 4.23) | 230  (197, 265) | 5.09  (4.37, 5.82) |
| Georgia | 237  (194, 306) | 3.82  (3.14, 4.93) | 360  (296, 427) | 6.17  (5.09, 7.37) |
| South Asia | 7582  (6135, 8935) | 1.42  (1.14, 1.68) | 38731  (34043, 43732) | 2.86  (2.51, 3.24) |
| Nepal | 93  (52, 138) | 1.04  (0.56, 1.62) | 583  (350, 851) | 2.76  (1.65, 4.08) |
| Bangladesh | 590  (373, 824) | 1.31  (0.81, 1.83) | 2616  (1652, 3879) | 2.07  (1.30, 3.09) |
| Pakistan | 815  (668, 958) | 1.47  (1.20, 1.73) | 3033  (2376, 3955) | 2.91  (2.27, 3.76) |
| India | 6081  (4969, 7205) | 1.44  (1.17, 1.70) | 32482  (28160, 37343) | 2.95  (2.55, 3.40) |
| Bhutan | 3  (2, 5) | 1.35  (0.67, 2.14) | 16  (9, 25) | 3.05  (1.75, 4.65) |
| Southeast Asia and Oceania | 5951  (5439, 6512) | 2.36  (2.17, 2.57) | 25376  (20339, 31451) | 4.32  (3.46, 5.34) |
| Viet Nam | 673  (551, 799) | 1.70  (1.41, 2.01) | 3825  (2972, 4870) | 4.30  (3.38, 5.40) |
| Sri Lanka | 179  (161, 198) | 1.71  (1.55, 1.88) | 717  (536, 953) | 2.86  (2.14, 3.77) |
| Malaysia | 162  (139, 188) | 1.83  (1.56, 2.16) | 928  (718, 1170) | 3.61  (2.81, 4.53) |
| Timor-Leste | 4  (3, 6) | 1.57  (1.06, 2.14) | 23  (16, 29) | 2.86  (2.05, 3.69) |
| Niue | 0  (0, 0) | 3.68  (2.87, 4.5) | 0  (0, 0) | 6.18  (4.67, 7.91) |
| Tonga | 2  (1, 2) | 2.79  (2.15, 3.53) | 3  (2, 4) | 4.26  (3.12, 5.61) |
| Maldives | 2  (2, 3) | 2.83  (2.01, 3.87) | 12  (10, 14) | 4.24  (3.49, 5.08) |
| Tuvalu | 0  (0, 0) | 2.49  (1.99, 3.01) | 0  (0, 1) | 3.83  (2.83, 5.13) |
| Myanmar | 531  (395, 709) | 2.29  (1.72, 3.03) | 1666  (1299, 2162) | 3.71  (2.91, 4.79) |
| Guam | 3  (2, 3) | 4.02  (3.39, 4.67) | 10  (8, 12) | 5.12  (4.25, 6.11) |
| Nauru | 0  (0, 0) | 4.14  (3.12, 5.46) | 0  (0, 0) | 5.84  (4.04, 7.89) |
| Cook Islands | 0  (0, 1) | 3.64  (2.96, 4.38) | 1  (1, 1) | 4.82  (4.05, 5.71) |
| Marshall Islands | 0  (0, 0) | 2.28  (1.90, 2.71) | 1  (1, 2) | 3.81  (2.83, 4.97) |
| Micronesia (Federated States of) | 1  (1, 2) | 3.07  (2.46, 3.80) | 3  (2, 5) | 5.06  (3.54, 6.81) |
| Thailand | 1119  (990, 1271) | 3.17  (2.81, 3.60) | 3999  (2986, 5156) | 3.95  (2.96, 5.09) |
| Palau | 1  (1, 1) | 7.91  (6.06, 10.22) | 2  (2, 3) | 11.43  (8.81, 14.37) |
| Samoa | 3  (3, 4) | 4.05  (3.36, 4.87) | 6  (5, 8) | 4.22  (3.4, 5.41) |
| American Samoa | 1  (1, 1) | 3.08  (2.56, 3.57) | 2  (2, 3) | 4.95  (4.10, 5.90) |
| Cambodia | 94  (70, 122) | 2.10  (1.56, 2.72) | 406  (323, 491) | 3.52  (2.82, 4.19) |
| Lao People's Democratic Republic | 48  (33, 67) | 2.27  (1.61, 3.14) | 140  (108, 180) | 3.31  (2.59, 4.14) |
| Fiji | 9  (7, 11) | 2.58  (2.11, 3.13) | 29  (23, 37) | 4.08  (3.22, 5.07) |
| Vanuatu | 1  (1, 2) | 1.80  (1.31, 2.46) | 5  (4, 7) | 2.95  (2.30, 3.84) |
| Papua New Guinea | 22  (14, 33) | 1.20  (0.76, 1.80) | 79  (55, 114) | 1.72  (1.21, 2.44) |
| Solomon Islands | 3  (2, 4) | 1.80  (1.31, 2.45) | 9  (6, 13) | 2.97  (2.06, 3.94) |
| Indonesia | 2151  (1783, 2511) | 2.22  (1.85, 2.58) | 10322  (6797, 14366) | 5.04  (3.30, 6.97) |
| Mauritius | 30  (28, 32) | 4.11  (3.81, 4.44) | 90  (72, 110) | 5.18  (4.16, 6.33) |
| Northern Mariana Islands | 1  (0, 1) | 3.40  (2.82, 4.18) | 4  (3, 4) | 7.49  (6.40, 8.69) |
| Philippines | 947  (841, 1062) | 3.20  (2.87, 3.56) | 3208  (2577, 3968) | 4.19  (3.39, 5.19) |
| Seychelles | 3  (3, 3) | 5.16  (4.54, 5.8) | 8  (7, 9) | 7.17  (6.26, 8.25) |
| Tokelau | 0  (0, 0) | 2.51  (1.86, 3.21) | 0  (0, 0) | 4.26  (3.00, 5.51) |
| Kiribati | 1  (1, 1) | 2.20  (1.78, 2.61) | 2  (1, 2) | 2.79  (2.11, 3.64) |
| Brunei Darussalam | 6  (5, 7) | 6.43  (5.47, 7.50) | 24  (21, 27) | 9.24  (8.03, 10.51) |
| Singapore | 115  (107, 124) | 5.44  (5.01, 5.86) | 463  (370, 572) | 6.07  (4.86, 7.53) |

**Appendix table 2: Deaths due to pancreatic cancer in 1990 and 2019 for both sexes in age-standardised rates by location.**

|  | 1990 | | 2019 | |
| --- | --- | --- | --- | --- |
|  | Counts (95% UI) | Rate (95% UI) | Counts (95% UI) | Rate (95% UI) |
| Asia | 18421  (15007, 23061) | 1.02  (0.83, 1.27) | 71303  (53638, 94337) | 1.55  (1.16, 2.06) |
| East Asia | 7381  (5595, 9777) | 0.89  (0.68, 1.17) | 37092  (27663, 49037) | 1.81  (1.34, 2.39) |
| Taiwan (Province of China) | 205  (167, 254) | 1.30  (1.05, 1.61) | 934  (615, 1410) | 2.33  (1.53, 3.51) |
| China | 7081  (5356, 9403) | 0.89  (0.68, 1.17) | 35848  (26641, 47589) | 1.81  (1.35, 2.4) |
| Republic of Korea | 720  (594, 866) | 2.53  (2.07, 3.07) | 2225  (1654, 2948) | 2.50  (1.85, 3.33) |
| Japan | 5644  (4826, 6674) | 3.31  (2.81, 3.92) | 10289  (7755, 13474) | 2.74  (2.15, 3.51) |
| Democratic People's Republic of Korea | 94  (63, 141) | 0.59  (0.40, 0.87) | 310  (210, 443) | 0.96  (0.66, 1.37) |
| Central Asia | 309  (240, 393) | 0.67  (0.52, 0.86) | 1254  (923, 1677) | 1.81  (1.32, 2.45) |
| Uzbekistan | 30  (20, 48) | 0.27  (0.18, 0.45) | 208  (138, 302) | 1.21  (0.79, 1.78) |
| Armenia | 56  (43, 72) | 2.10  (1.60, 2.74) | 151  (107, 202) | 3.62  (2.57, 4.84) |
| Mongolia | 7  (5, 10) | 0.71  (0.53, 0.94) | 35  (24, 48) | 1.59  (1.12, 2.16) |
| Turkmenistan | 7  (5, 9) | 0.38  (0.29, 0.47) | 40  (26, 57) | 1.06  (0.70, 1.52) |
| Kazakhstan | 42  (31, 54) | 0.34  (0.25, 0.45) | 375  (268, 512) | 2.18  (1.54, 3.00) |
| Azerbaijan | 45  (33, 59) | 0.90  (0.67, 1.20) | 203  (142, 276) | 2.35  (1.64, 3.24) |
| Tajikistan | 23  (14, 36) | 0.85  (0.51, 1.37) | 51  (32, 78) | 1.28  (0.79, 1.95) |
| Kyrgyzstan | 32  (25, 40) | 1.05  (0.84, 1.30) | 67  (50, 88) | 1.50  (1.13, 1.98) |
| Georgia | 68  (50, 90) | 1.08  (0.79, 1.44) | 125  (88, 170) | 2.11  (1.49, 2.88) |
| South Asia | 1756  (1149, 2559) | 0.36  (0.24, 0.53) | 9359  (6117, 13993) | 0.72  (0.47, 1.08) |
| Nepal | 26  (13, 43) | 0.34  (0.17, 0.57) | 180  (97, 293) | 0.91  (0.49, 1.50) |
| Bangladesh | 164  (90, 256) | 0.39  (0.21, 0.60) | 674  (365, 1150) | 0.55  (0.30, 0.94) |
| Pakistan | 244  (179, 330) | 0.46  (0.34, 0.62) | 807  (509, 1229) | 0.84  (0.53, 1.28) |
| India | 1321  (850, 1944) | 0.35  (0.22, 0.52) | 7695  (4915, 11678) | 0.73  (0.46, 1.11) |
| Bhutan | 1  (0, 1) | 0.25  (0.11, 0.46) | 3  (2, 6) | 0.67  (0.31, 1.23) |
| Southeast Asia and Oceania | 1517  (1196, 1925) | 0.65  (0.51, 0.83) | 6967  (4805, 10113) | 1.24  (0.85, 1.81) |
| Viet Nam | 150  (105, 208) | 0.39  (0.27, 0.54) | 971  (629, 1444) | 1.14  (0.73, 1.72) |
| Sri Lanka | 45  (30, 63) | 0.47  (0.32, 0.66) | 205  (112, 352) | 0.84  (0.46, 1.44) |
| Malaysia | 44  (32, 61) | 0.53  (0.37, 0.74) | 274  (178, 411) | 1.11  (0.72, 1.68) |
| Timor-Leste | 1  (1, 1) | 0.39  (0.21, 0.62) | 6  (3, 9) | 0.76  (0.44, 1.17) |
| Niue | 0  (0, 0) | 1.25  (0.80, 1.85) | 0  (0, 0) | 2.39  (1.44, 3.69) |
| Tonga | 1  (0, 1) | 1.1  (0.73, 1.53) | 1  (1, 2) | 1.72  (1.08, 2.55) |
| Maldives | 1  (0, 1) | 0.86  (0.55, 1.34) | 3  (2, 5) | 1.30  (0.89, 1.84) |
| Tuvalu | 0  (0, 0) | 0.82  (0.57, 1.15) | 0  (0, 0) | 1.42  (0.86, 2.23) |
| Myanmar | 187  (127, 260) | 0.88  (0.61, 1.20) | 517  (350, 750) | 1.24  (0.84, 1.81) |
| Guam | 1  (1, 1) | 1.26  (0.87, 1.76) | 3  (2, 5) | 1.70  (1.14, 2.45) |
| Nauru | 0  (0, 0) | 1.52  (1.02, 2.16) | 0  (0, 0) | 2.32  (1.39, 3.53) |
| Cook Islands | 0  (0, 0) | 1.30  (0.88, 1.84) | 0  (0, 1) | 1.87  (1.24, 2.71) |
| Marshall Islands | 0  (0, 0) | 0.71  (0.44, 1.09) | 0  (0, 1) | 1.39  (0.76, 2.29) |
| Micronesia (Federated States of) | 1  (0, 1) | 1.12  (0.78, 1.54) | 1  (1, 2) | 2.08  (1.25, 3.11) |
| Thailand | 330  (259, 419) | 1.00  (0.79, 1.28) | 1128  (723, 1739) | 1.12  (0.72, 1.73) |
| Palau | 0  (0, 0) | 2.37  (1.45, 3.65) | 1  (0, 1) | 3.88  (2.26, 6.10) |
| Samoa | 1  (1, 2) | 1.58  (1.17, 2.17) | 2  (2, 4) | 1.77  (1.22, 2.54) |
| American Samoa | 0  (0, 0) | 1.31  (0.90, 1.83) | 1  (1, 1) | 2.23  (1.46, 3.23) |
| Cambodia | 27  (17, 39) | 0.66  (0.43, 0.94) | 123  (88, 171) | 1.16  (0.83, 1.58) |
| Lao People's Democratic Republic | 14  (9, 22) | 0.72  (0.46, 1.11) | 41  (27, 60) | 1.06  (0.71, 1.55) |
| Fiji | 3  (2, 5) | 1.05  (0.72, 1.49) | 11  (7, 18) | 1.66  (1.02, 2.57) |
| Vanuatu | 0  (0, 0) | 0.51  (0.32, 0.78) | 1  (1, 2) | 0.88  (0.52, 1.47) |
| Papua New Guinea | 6  (3, 10) | 0.35  (0.19, 0.60) | 22  (13, 38) | 0.52  (0.30, 0.88) |
| Solomon Islands | 1  (0, 1) | 0.60  (0.40, 0.89) | 3  (2, 5) | 1.07  (0.68, 1.60) |
| Indonesia | 426  (304, 580) | 0.48  (0.34, 0.65) | 2690  (1573, 4347) | 1.38  (0.80, 2.26) |
| Mauritius | 8  (6, 11) | 1.16  (0.82, 1.58) | 29  (18, 47) | 1.7  (1.03, 2.70) |
| Northern Mariana Islands | 0  (0, 0) | 1.22  (0.86, 1.69) | 1  (1, 2) | 2.71  (1.84, 3.83) |
| Philippines | 282  (219, 359) | 1.06  (0.82, 1.35) | 967  (692, 1325) | 1.37  (0.98, 1.85) |
| Seychelles | 1  (1, 1) | 1.51  (1.10, 2.03) | 3  (2, 4) | 2.56  (1.7, 3.71) |
| Tokelau | 0  (0, 0) | 0.80  (0.51, 1.14) | 0  (0, 0) | 1.53  (0.91, 2.37) |
| Kiribati | 0  (0, 0) | 0.96  (0.73, 1.28) | 1  (1, 1) | 1.40  (0.97, 1.97) |
| Brunei Darussalam | 2  (2, 3) | 2.72  (1.87, 3.81) | 8  (5, 12) | 3.32  (2.14, 4.89) |
| Singapore | 33  (25, 44) | 1.59  (1.18, 2.12) | 107  (73, 154) | 1.40  (0.95, 2.02) |

**Appendix table 3: DALYs due to pancreatic cancer in 1990 and 2019 for both sexes in age-standardised rates by location.**

|  | 1990 | | 2019 | |
| --- | --- | --- | --- | --- |
|  | Counts (95% UI) | Rate (95% UI) | Counts (95% UI) | Rate (95% UI) |
| Asia | 441609  (355797, 559680) | 21.59  (17.51, 27.12) | 1576951  (1199781, 2085291) | 34.63  (26.34, 45.79) |
| East Asia | 189242  (139869, 253651) | 20.54  (15.46, 27.36) | 856283  (631770, 1142736) | 58.16  (42.91, 77.62) |
| Taiwan (Province of China) | 5343  (4368, 6573) | 31.43  (25.72, 38.66) | 21098  (14094, 31773) | 89.32  (59.67, 134.52) |
| China | 181258  (133618, 243797) | 20.44  (15.34, 27.31) | 827266  (608036, 1103963) | 58.16  (42.75, 77.62) |
| Republic of Korea | 18928  (15669, 22804) | 58.98  (48.72, 71.13) | 44531  (33541, 58314) | 83.39  (62.81, 109.21) |
| Japan | 120330  (103259, 140872) | 69.37  (59.54, 81.36) | 175010  (137889, 222905) | 136.95  (107.90, 174.43) |
| Democratic People's Republic of Korea | 2642  (1727, 4023) | 14.83  (9.84, 22.32) | 7919  (5219, 11442) | 30.19  (19.89, 43.62) |
| Central Asia | 8014  (6278, 10041) | 16.46  (12.93, 20.68) | 32186  (23850, 42695) | 34.41  (25.5, 45.65) |
| Uzbekistan | 766  (515, 1171) | 6.54  (4.41, 10.16) | 5666  (3798, 8213) | 16.82  (11.28, 24.39) |
| Armenia | 1456  (1133, 1876) | 50.57  (39.02, 65.00) | 3409  (2450, 4568) | 112.90  (81.13, 151.29) |
| Mongolia | 179  (129, 243) | 16.77  (12.19, 22.51) | 962  (637, 1369) | 28.40  (18.79, 40.41) |
| Turkmenistan | 178  (141, 219) | 8.96  (7.04, 11.12) | 1053  (708, 1506) | 20.71  (13.93, 29.63) |
| Kazakhstan | 1027  (774, 1332) | 7.92  (5.96, 10.28) | 9706  (7021, 13063) | 52.77  (38.17, 71.03) |
| Azerbaijan | 1228  (921, 1636) | 23.18  (17.42, 30.44) | 5363  (3717, 7282) | 52.17  (36.17, 70.84) |
| Tajikistan | 537  (354, 794) | 19.36  (12.53, 28.97) | 1287  (839, 1956) | 13.56  (8.84, 20.61) |
| Kyrgyzstan | 854  (685, 1062) | 27.44  (22.07, 34.19) | 1753  (1319, 2326) | 26.83  (20.19, 35.59) |
| Georgia | 1789  (1332, 2369) | 27.63  (20.74, 36.62) | 2988  (2100, 4037) | 81.54  (57.29, 110.15) |
| South Asia | 42994  (27986, 62600) | 7.67  (5.01, 11.20) | 211797  (138268, 313331) | 11.73  (7.66, 17.36) |
| Nepal | 609  (316, 989) | 6.78  (3.44, 11.09) | 3804  (2032, 6261) | 12.51  (6.68, 20.58) |
| Bangladesh | 3851  (2098, 6098) | 8.37  (4.55, 13.23) | 14548  (7870, 24848) | 9.13  (4.94, 15.60) |
| Pakistan | 5461  (3973, 7340) | 9.67  (7.12, 12.99) | 19414  (12010, 29689) | 8.66  (5.36, 13.25) |
| India | 33060  (21201, 48695) | 7.34  (4.74, 10.83) | 173956  (109789, 264119) | 12.51  (7.89, 18.99) |
| Bhutan | 13  (6, 24) | 5.32  (2.32, 9.75) | 75  (34, 140) | 9.93  (4.55, 18.61) |
| Southeast Asia and Oceania | 38084  (29514, 48596) | 14.50  (11.37, 18.41) | 162914  (112186, 235516) | 24.18  (16.65, 34.95) |
| Viet Nam | 3458  (2394, 4865) | 8.53  (5.95, 11.97) | 22104  (14588, 32440) | 22.94  (15.14, 33.66) |
| Sri Lanka | 1042  (700, 1451) | 9.67  (6.52, 13.44) | 4321  (2363, 7397) | 19.77  (10.81, 33.85) |
| Malaysia | 1059  (760, 1444) | 11.51  (8.23, 15.76) | 6216  (4039, 9286) | 19.86  (12.90, 29.67) |
| Timor-Leste | 25  (13, 41) | 8.60  (4.69, 13.73) | 133  (75, 202) | 9.93  (5.64, 15.12) |
| Niue | 1  (0, 1) | 28.91  (18.41, 42.72) | 1  (1, 2) | 70.44  (42.42, 107.90) |
| Tonga | 14  (9, 19) | 24.26  (15.93, 33.90) | 30  (19, 45) | 29.36  (18.14, 43.85) |
| Maldives | 16  (9, 26) | 17.93  (10.91, 28.59) | 74  (53, 104) | 14.89  (10.54, 20.78) |
| Tuvalu | 1  (1, 2) | 19.49  (13.50, 27.49) | 3  (2, 5) | 29.53  (17.71, 45.95) |
| Myanmar | 4624  (3062, 6700) | 19.55  (13.27, 27.78) | 11538  (7655, 17054) | 21.10  (14.00, 31.19) |
| Guam | 23  (16, 31) | 28.06  (19.53, 38.44) | 78  (53, 112) | 45.66  (31.26, 65.46) |
| Nauru | 1  (1, 2) | 36.32  (24.00, 52.02) | 3  (1, 4) | 24.71  (13.98, 38.85) |
| Cook Islands | 4  (3, 6) | 29.78  (19.96, 42.21) | 11  (7, 15) | 58.82  (38.99, 85.78) |
| Marshall Islands | 3  (2, 4) | 16.84  (10.73, 25.70) | 12  (7, 21) | 21.83  (11.94, 36.34) |
| Micronesia (Federated States of) | 13  (9, 19) | 27.11  (18.55, 37.72) | 38  (22, 59) | 37.38  (21.75, 57.39) |
| Thailand | 8400  (6520, 10651) | 22.59  (17.68, 28.73) | 24023  (15374, 37110) | 34.26  (21.93, 52.93) |
| Palau | 5  (3, 8) | 53.55  (33.65, 80.82) | 19  (11, 29) | 105.04  (62.65, 163.46) |
| Samoa | 34  (24, 47) | 37.51  (27.14, 51.94) | 62  (42, 90) | 29.11  (19.78, 42.75) |
| American Samoa | 7  (5, 10) | 29.99  (20.72, 41.66) | 26  (17, 37) | 46.03  (30.10, 66.67) |
| Cambodia | 668  (429, 1000) | 14.74  (9.56, 21.50) | 2859  (2007, 4012) | 17.22  (12.09, 24.16) |
| Lao People's Democratic Republic | 364  (227, 580) | 16.79  (10.63, 26.25) | 995  (660, 1463) | 13.91  (9.22, 20.44) |
| Fiji | 89  (61, 124) | 23.77  (16.46, 33.49) | 284  (174, 442) | 31.18  (19.13, 48.47) |
| Vanuatu | 8  (5, 12) | 11.40  (6.95, 17.71) | 36  (21, 61) | 12.19  (7.04, 20.60) |
| Papua New Guinea | 163  (87, 282) | 8.26  (4.48, 14.19) | 614  (343, 1057) | 6.23  (3.48, 10.72) |
| Solomon Islands | 23  (14, 35) | 15  (9.39, 22.65) | 89  (52, 140) | 13.59  (7.9, 21.39) |
| Indonesia | 11058  (7856, 15041) | 10.80  (7.69, 14.72) | 66259  (39728, 106628) | 25.54  (15.31, 41.1) |
| Mauritius | 202  (146, 272) | 26.50  (19.13, 35.85) | 671  (404, 1066) | 52.54  (31.66, 83.5) |
| Northern Mariana Islands | 5  (4, 8) | 27.85  (19.39, 38.82) | 35  (23, 49) | 81.57  (54.81, 115.40) |
| Philippines | 7098  (5478, 8968) | 23.07  (17.85, 29.39) | 23445  (16637, 32030) | 20.91  (14.84, 28.56) |
| Seychelles | 19  (14, 26) | 34.74  (25.15, 46.24) | 62  (42, 89) | 61.06  (41.38, 87.54) |
| Tokelau | 0  (0, 0) | 18.70  (11.9, 26.36) | 0  (0, 1) | 33.60  (19.90, 51.55) |
| Kiribati | 9  (6, 11) | 22.35  (16.64, 29.88) | 21  (14, 31) | 17.98  (11.93, 25.99) |
| Brunei Darussalam | 54  (37, 75) | 56.87  (39.22, 79.62) | 192  (125, 284) | 43.85  (28.66, 65.02) |
| Singapore | 794  (601, 1046) | 34.99  (26.41, 46.26) | 2279  (1575, 3229) | 40.20  (27.79, 56.98) |


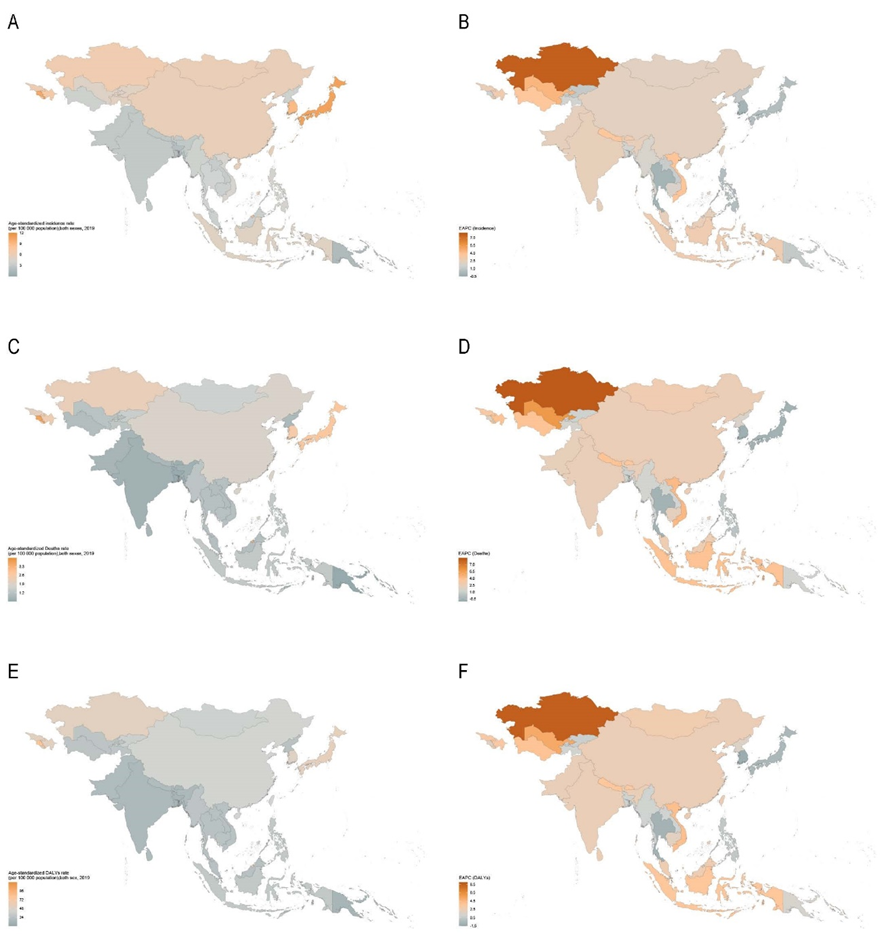


Appendix Figure 1: **Map of age-standardized incidence rate, mortality rate, DALY rate of pancreatic cancer in Asia, 2019, and age-standardized Estimated Annual Percentage Change from 1990 to 2019.** (A) Map of age-standardized incidence rate in Asia, 2019. (B) EAPC of age-standardized incidence rate from 1990 to 2019. (C) Map of age-standardized mortality rate in Asia, 2019. (D) EAPC of age-standardized mortality rate from 1990 to 2019. (E) Map of age-standardized DALY rate in Asia, 2019. (F) EAPC of age-standardized DALY rate from 1990 to 2019.


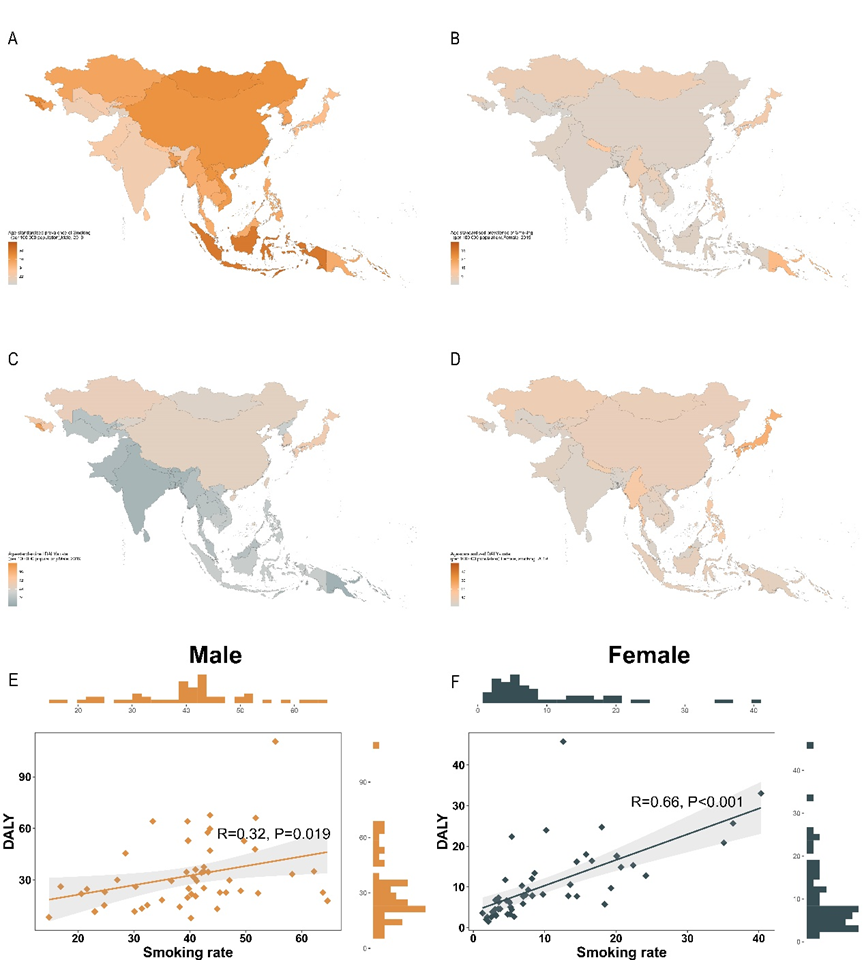


Appendix Figure 2: **Map of the smoking prevalence of pancreatic cancer across sex in Asia, 2019 and age-standardized DALY rate of pancreatic cancer due to smoking, 2019 and their correlation.** (A) Map of smoking prevalence among males in Asia, 2019. (B) Map of smoking prevalence among females in Asia, 2019 (C) Age-standardized DALY rate of pancreatic cancer among males in Asia, 2019. (D) Age-standardized DALY rate of pancreatic cancer among females in Asia, 2019.
